# Moon Regolith Simulant-Based All-3D-Printed Triboelectric Nanogenerator for Effective Mechanical Energy Conversion

**DOI:** 10.1021/acs.energyfuels.5c04047

**Published:** 2026-02-04

**Authors:** Alex Yohannan, Keval K. Sonigara, Jayraj V. Vaghasiya, Martin Pumera

**Affiliations:** † Future Energy and Innovation Laboratory, Central European Institute of Technology, 48274 Brno University of Technology, Purkyňova 123, Brno 61200, Czech Republic; ‡ School of Biomedical Engineering, Faculty of Engineering, The University of Sydney, Sydney, NSW 2008, Australia; § Faculty of Electrical Engineering and Computer Science, VSB - Technical University of Ostrava, 17. listopadu 2172/15, 70800 Ostrava, Czech Republic; ∥ Department of Medical Research, China Medical University Hospital, China Medical University, No. 91 Hsueh-Shih Road, Taichung 40402, Taiwan; ⊥ Department of Chemical and Biomolecular Engineering, Yonsei University, 50 Yonsei-ro, Seodaemun-gu, Seoul 03722, Korea

## Abstract

Sustained human activity on the Moon will require energy systems that can be manufactured directly from lunar materials, avoiding the mass and cost constraints of transporting devices from the Earth. Here, we demonstrate a triboelectric nanogenerator (TENG) fabricated using a lunar regolith (LR) simulant as an active triboelectric component through a scalable 3D-printing strategy. LR incorporation significantly enhances charge generation in LR/PLA (poly­(lactic acid)) composite electrodes by modifying surface properties and increasing effective contact electrification. Multiple electrode architectures were evaluated to optimize performance, with the best design delivering an open-circuit voltage of ∼17.4 (±0.4) V and a short-circuit current of ∼0.96 (±0.2) μA at 10 Hz for a 20 × 30 mm device. The prototype is capable of real-scale power demonstrations, validating its practical utility. This work introduces a viable route for in situ resource utilization and 3D printing to establish LR-based triboelectric devices as a promising approach for energy autonomy in future lunar habitats.

## Graphical abstract

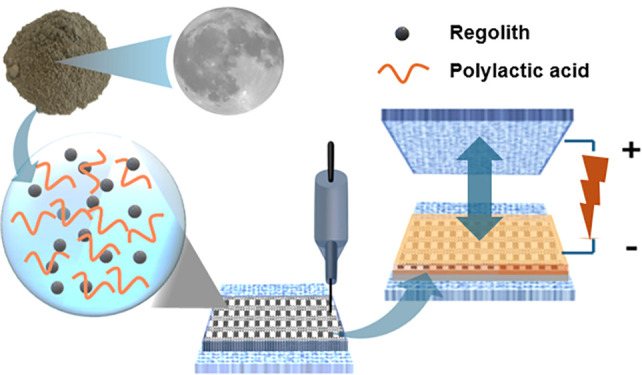

## 1 Introduction

The space agencies are ready to take a futuristic step toward exploration beyond the earth, focusing more on the search for sustainable human settlement on the lunar and Mars environment.^1,2^ The Artemis Program,^3^ spearheaded by the National Aeronautics and Space Administration (NASA), is expected to create further human explorations of the Moon to build a permanent base, which will facilitate long-distance expeditions through the development of the Artemis Base Camp.^3^ Given the necessity of meeting daily energy demands, recent studies have focused on developing power generation and energy storage systems utilizing LR simulants as a resource.^4,5^ In Situ Resource Utilization (ISRU) helps reduce the amount of mass that must be launched from the Earth and also brings down the expenses incurred from launching a series of missions, thereby making it possible to increase the duration of the mission.^6–8^ The LR is found to have a greater variety of minerals. More specifically, LR consists predominantly of aluminum, silicon, calcium, iron, sodium, and titanium oxides^9^ making it a valuable resource for developing energy generation and storage systems for lunar missions.

Energy storage and harvesting for lunar exploration have evolved significantly over these decades, driven mainly by the surmounting of challenges uniquely offered by the lunar environment. Early exploration into this domain identified LR as a source of thermal energy storage.^10^ Pioneering experiments were performed by Richter et al.^10^ to study the feasibility of lunar soil as a thermal energy storage medium.^10^ This foundational research emphasizes the potential of using in situ materials to support sustained missions on the lunar surface. Based on that, Wegeng et al.^11^ demonstrated that a thermal energy reservoir, which could be artificially created from processed LR, could store heat and supplemental electrical power throughout the long lunar nights. At the same time, Fleth et al.^12^ modeled a system in which treated LR was combined with a thermoelectric generator. Although this approach was less effective than conventional power technologies, it represents an essential step toward innovative, resource-efficient solutions for lunar energy harvesting. In recent years, developments have expanded beyond energy storage to include infrastructure development and the use of solar energy.^10,13^ It has previously been reported that it is possible to obtain components for solar power systems from the Moon, as more than 90% of the materials required for solar cells can be found on the Moon.^14,15^ Earlier, Duke et al.^16^ proposed an ingenious fabrication technique for producing silicon photovoltaic cells directly on the lunar regolith using concentrated solar energy. This approach could substantially reduce the cost of energy production on the Moon, thereby enabling efficient propellant production and supporting sustained human settlement. Recent studies have explored advanced in situ fabrication techniques using lunar regolith simulants, including high-pressure extrusion of regolith/polymer composites,^17,18^ solar or xenon-light sintering,^13,19^ and microwave-assisted consolidation for structural components.^20,21^ While these efforts have largely focused on construction or solar power substrates,^15,22^ their underlying principles highlight the potential of regolith as a functional material for energy conversion devices.

However, fabricating such devices is very challenging because of extended lunar nights, low atmospheric pressure, extreme temperature variations, dust accumulation, and low gravitational fields. These limitations restrict the use of materials available on the moon or future space sites. Hence, alternative energy-harvesting systems and fabrication methods need to be developed from resources like LR. Recent studies have demonstrated that the combination of ISRU and additive manufacturing (AM) can facilitate the development of the power generation equipment on the Moon.^23–25^ The 3D printing now provides a transformative methodology for the fabrication of complex structures on-site using ISRU feedstock. The International Space Station (ISS) is already using a fused deposition modeling (FDM) 3D printer in the space environment to print hardware parts.^26^ Hence, energy generation devices fabricated by FDM printing and utilizing LR can provide possible solutions to existing limitations. This method not only reduces the need for imports from the Earth but also contributes to the sustainability of long-duration missions. Our approach extends these concepts to triboelectric nanogenerator development, integrating LR with PLA in a 3D-printable filament, thereby demonstrating a novel pathway for small-scale, mechanically robust energy harvesters compatible with lunar resource constraints.

Among various energy conversion technologies, TENGs have emerged as a flexible means to convert mechanical energy into electricity. Since their invention, TENGs have rapidly advanced from simple, prototype-stage devices tested in laboratories to highly efficient, multifunctional devices applicable across a wide range of fields.^27,28^ Its ability to function in resource-constrained environments made it a perfect candidate for extraterrestrial missions, where abundant mechanical vibration and triboelectric effects are realized. TENGs offer several advantages that make them particularly suited to lunar energy harvesting. Unlike thermal energy storage systems, which rely on heat retention and are limited during long lunar nights, or solar-based systems, which require continuous sunlight and are impacted by dust accumulation and extreme temperature variations, TENGs can directly convert mechanical energy from environmental vibrations, astronaut movement, or robotic operations into electricity. Their simple architecture and flexibility allow robust operation under low-gravity conditions and in vacuum environments, while their tolerance to dust makes them resilient to lunar surface conditions. These characteristics, combined with the ability to fabricate small-scale, lightweight devices, make TENGs ideal candidates for in situ energy harvesting on the Moon.

This work proposes an advanced approach to fabricating TENG devices using LR for the first time as a triboelectric component. We designed 3D-printed TENG devices that maximize energy-harvesting capabilities by combining LR with PLA in a printable filament. The well-dispersed LR in PLA and multimaterial printed architecture further enhance charge generation and collection.^29^ This approach not only provides a renewable energy source but also proves the compatibility of cosmic simulants with TENG-based energy generation mechanisms. Herein, we report on the structural design, fabrication process, and performance analysis of a fully 3D-printed TENG device using the moon LR simulant as an important component. The experimental measurements have shown a peak *V*_oc_ of ∼17.4 (±1) V and an *I*_sc_ of ∼0.96 (±0.2) μA for a 20 mm × 30 mm device, thereby proving the operability of the devices for self-powering tiny gadgets integrated with space suits or sensors. The work has bridged gaps in ISRU, 3D printing, and energy harvesting, providing a compelling step toward achieving sustainable lunar energy systems that enable long-term space exploration missions.

## 2 Results and Discussion

### 2.1 Fabrication and Characterization of LR-Based 3D Electrodes

Figure 1 introduces the overall concept of using a lunar regolith simulant as the triboelectric component in an entirely 3D-printed TENG device. Figure 1a shows the fabrication route for an FDM (fused deposition modeling) printable LR/PLA filament, together with digital images of the LR powder and PLA pellets. Although the LR/PLA filament used in this work was commercially sourced, the schematic is included to illustrate the composition and extrusion process that produce the composite. PLA was chosen as the printing matrix because it is widely used in FDM printing and is therefore well suited for reliable device fabrication.^30^ While PLA can exhibit weak piezoelectricity in its l-isomer form, it also readily accepts electrons from metals, resulting in favorable positive or negative triboelectric behavior depending on the pairing.^31^ As a result, PLA has become a common material in TENG studies, and its triboelectric properties are well-documented in previous reports.^32,33^

**Figure 1:**
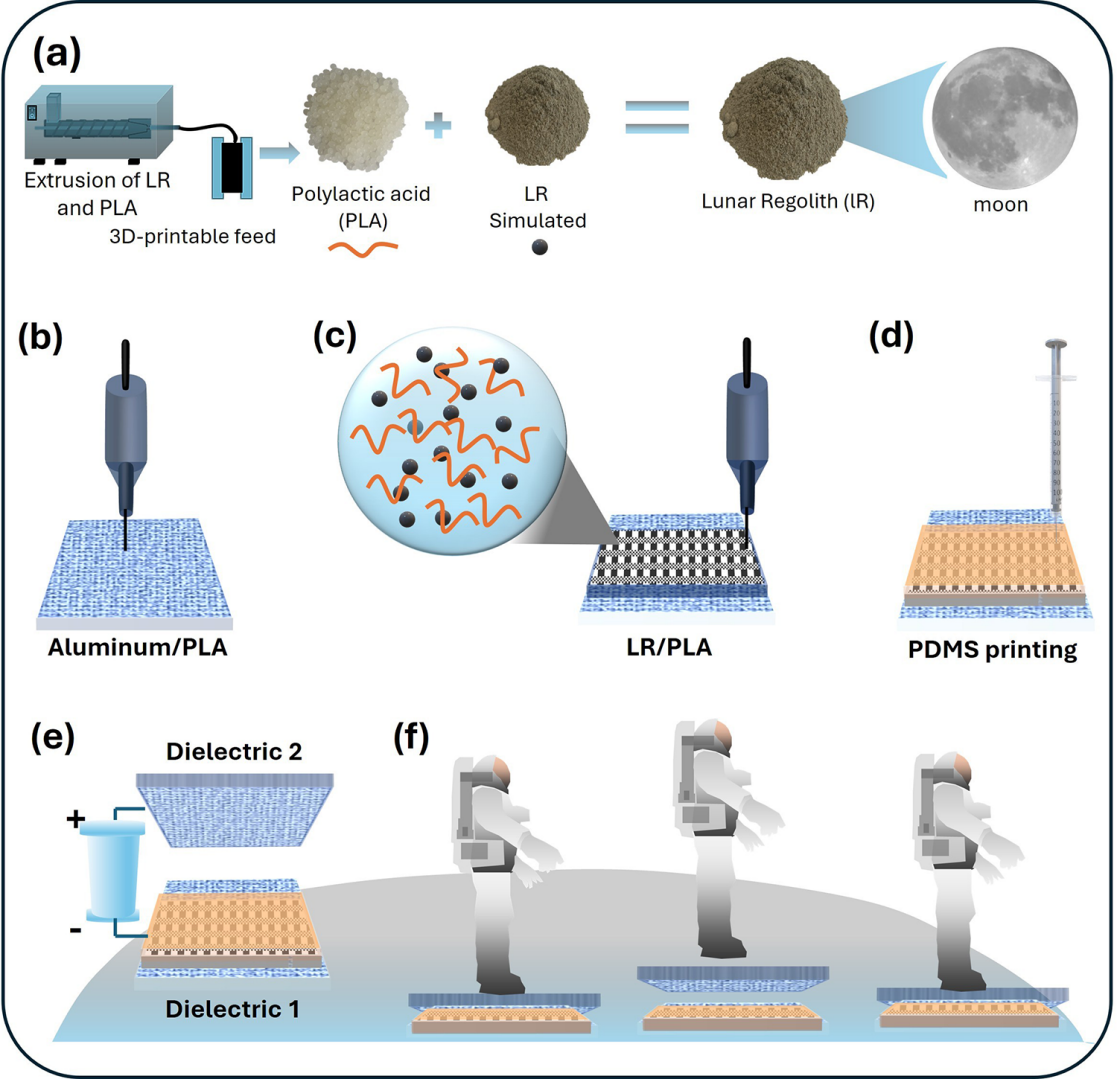
Schematic illustration of LR/PLA composite preparation, 3D-printing steps, and conceptual application of the all-3D-printed TENG. (a) Preparation of the LR/PLA composite filament, where PLA pellets and lunar regolith simulant powder are blended and extruded into a printable filament. (b) Multimaterial 3D-printing process showing deposition of the aluminum/PLA layer. (c) Printing of the patterned LR/PLA structure—cross-sectional schematic highlighting the LR particles dispersed within the PLA matrix. (d) Direct-ink-writing of PDMS into the patterned LR/PLA. (e) Assembly of the TENG device with dielectric 1 and 2. (f) Conceptual demonstration of the TENG integrated with astronaut jumping for energy harvesting.

Figure 1b–d outlines the stepwise fabrication of the multilayer dielectric 1. The process begins with printing the aluminum/PLA layer (Figure 1b), followed by printing the patterned LR/PLA composite directly onto the top of it (Figure 1c). Finally, PDMS (poly­(dimethylsiloxane)) is infused into the porous LR/PLA architecture to form the completed triboelectric electrode (Figure 1d). A digital photograph of the fully printed dielectric 1 is provided in Figure S1, showing its overall geometry, the printed mesh structure, and the PDMS-coated surface. The roughened appearance arises from the inherent porosity of the LR/PLA layer, which is preserved after PDMS infiltration. The electrode features are designed to increase the effective contact area and enhance charge generation during triboelectric operation. The details about dimensions, thickness, design protocol, and printing parameters are provided in Section 4. The functional TENG device was prepared by pairing dielectric 1 with dielectric 2, using an aluminum counter electrode coated on a 3D-printed PLA support in contact–separation mode (Figure 1e). The bottom panel (Figure 1f) illustrates a potential use case where the printed TENG harvests mechanical energy from an astronaut’s step on a TENG device and also shows the contact and separation events of two dielectric electrodes that generate electricity by harvesting the static charge. It also indicates the usefulness of the TENG device, which can operate when astronauts walk through jumping on it. It could generate electricityduring this operation and discharge the static charges on astronaut suits, usually accumulated in a drymoon environment.^34^ This example is included to show a possible future application of the LR-based TENG, highlighting how such devices could support ISRU strategies in space missions.

Before evaluating the device performance, we first examined the fundamental material characteristics of the LR/PLA composite and the 3D-printed architecture. These basic structural, morphological and surface properties were analyzed using X-ray diffraction (XRD), surface potential measurements, scanning electron microscopy (SEM), and energy-dispersive X-ray spectroscopy (EDX). The XRD was conducted to study qualitative composition and crystalline structures present in the composite (Figure 2a). The diffraction peaks are relevant to various minerals, majorly anorthosite, augite, ilmenite, and olivine. Among these, anorthosite and ilmenite match with the supplier information and others could be part of the basalt composition. Particularly, it reveals the presence of various oxides of silicate minerals in significant proportions, where the most intense peaks correspond to anorthite (CaAl_2_Si_2_O_8_), in agreement with the standard pattern for anorthite (JCPDS 00-041-1486) and with previous reports on anorthite-bearing glass–ceramics and regolith analogues. Also, refractions are observed around the 2theta values of 21.2°, 31.6°, and 42.3°, corresponding to the planes of (100), (110), and (111), indicating the presence of silica and other silicate-based minerals typical of lunar soil.^35,36^ Additional peaks are assigned to olivine (Mg_1.460_Fe_0.540_SiO_4_, JCPDS 01-076-0553) and clinopyroxene/augite (Ca (Mg, Fe) Si_2_O_6_, JCPDS 00-024-0203), which are the typical mafic silicate components of basaltic regolith.^37^ The XRD pattern also aligned to peaks corresponding to iron- and titanium-based oxides such as FeTiO_3_ (Ilmenite, JCPSD 01-075-1207). Furthermore, the filament revealed sharp and well-defined peaks at 16.59° and 19.02°, confirming the crystalline nature of PLA.^38^ This analysis confirms a fine physical blend of both materials. For the sake of extensiveness, the XRD diffraction of the virgin PLA filament used in this work is also provided in Supplementary Figure S2. This reference pattern is included to distinguish the characteristic crystalline peaks of PLA from those originating from the LR/PLA composite.

**Figure 2:**
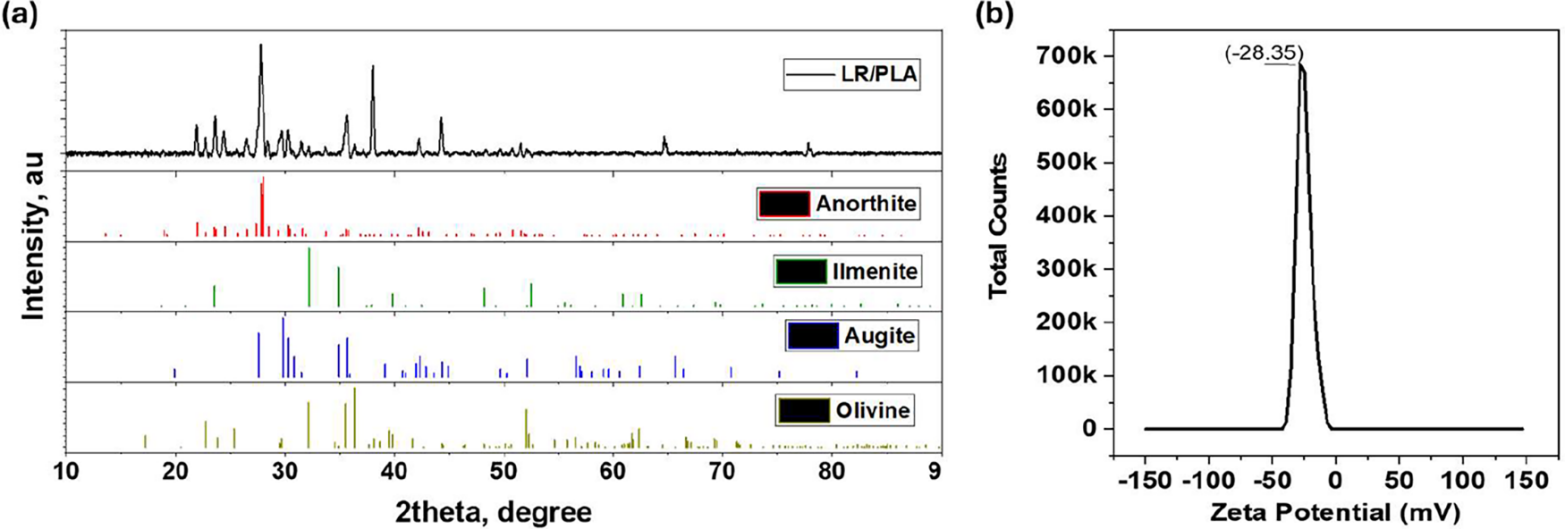
Structural analysis of LR/PLA. (a) XRD spectra and comparison with the best-match minerals in the composite: anorthite (JCPDS 00-041-1486), ilmenite (JSCPSD 01-075-1207), augite (JCPDS 00-024-0203), and olivine (JCPDS 01-076-0553). (b) Surface charge analysis.

The electronegative properties of the LR/PLA filament are defined through the surface charge measured using a zeta sizer, which yielded a zeta potential of −28.35 mV (Figure 2b). This negative charge is highly beneficial for the TENG applications as a negative dielectric component, as it significantly enhances the material’s ability to generate electrical energy through contact electrification. Here, the electronegativity of the LR/PLA 3D-printed porous structure has a greater ability to attract and retain charges on the surface. The zeta potential result confirms the suitability of LR for creating the TENG dielectric electrode.

To confirm the initial morphology of the LR/PLA filament prior to 3D printing, SEM analysis was carried out on the as-received filament, as shown in Supplementary Figure S3. The low-magnification image (Figure S3a) reveals a continuous filament structure with a rough surface and continuous porosity along its surface. The higher-magnification micrograph (Figure S3b) shows evenly distributed micropores and embedded particulate features within the PLA matrix, confirming the homogeneous dispersion of LR particles throughout the filament. These pores originate from the irregular shapes and surface asperities of the LR particles.

To confirm the effective multilateral 3D-printing of the dielectric 1 electrode, SEM and EDX were carried out across the section of layers. Figure 3a shows the cross-sectional SEM image of the transition across the PDMS, LR/PLA, and aluminum/PLA layers from top to bottom. A higher-magnification image clearly displays a well-fused boundary between the PDMS, LR/PLA composite, and the aluminum/PLA layer, without visible gaps or delamination (Figure 3b). This continuous interface confirms the strong adhesion formed during the successful FDM process and supports the mechanical stability of the device during repeated contact–separation cycles. Figure 3c shows a focused SEM image of the selected LR/PLA region, which exhibits a heterogeneous microstructure with embedded mineral particles distributed throughout the polymer matrix. The EDX spectrum and elemental mapping of this selected LR/PLA image reveal characteristic peaks of C and O originating from the PLA matrix,^39,40^ together with Al, Si, Fe, Ca, Mg, Na, Ti, and K, associated with the oxide-rich lunar regolith simulant^41,42^ (Figures S4 and S5). The full quantitative weight-percentage values obtained from the mapped region are provided in the Supporting Information (Table S1), showing that the composite contains a substantial inorganic fraction embedded within the polymer phase. The dominance of silicate- and aluminosilicate-based elements is consistent with the mineralogical signatures of widely used regolith analogues,^43,44^ supporting the heterogeneous particle distribution observed in the SEM micrographs.

**Figure 3:**
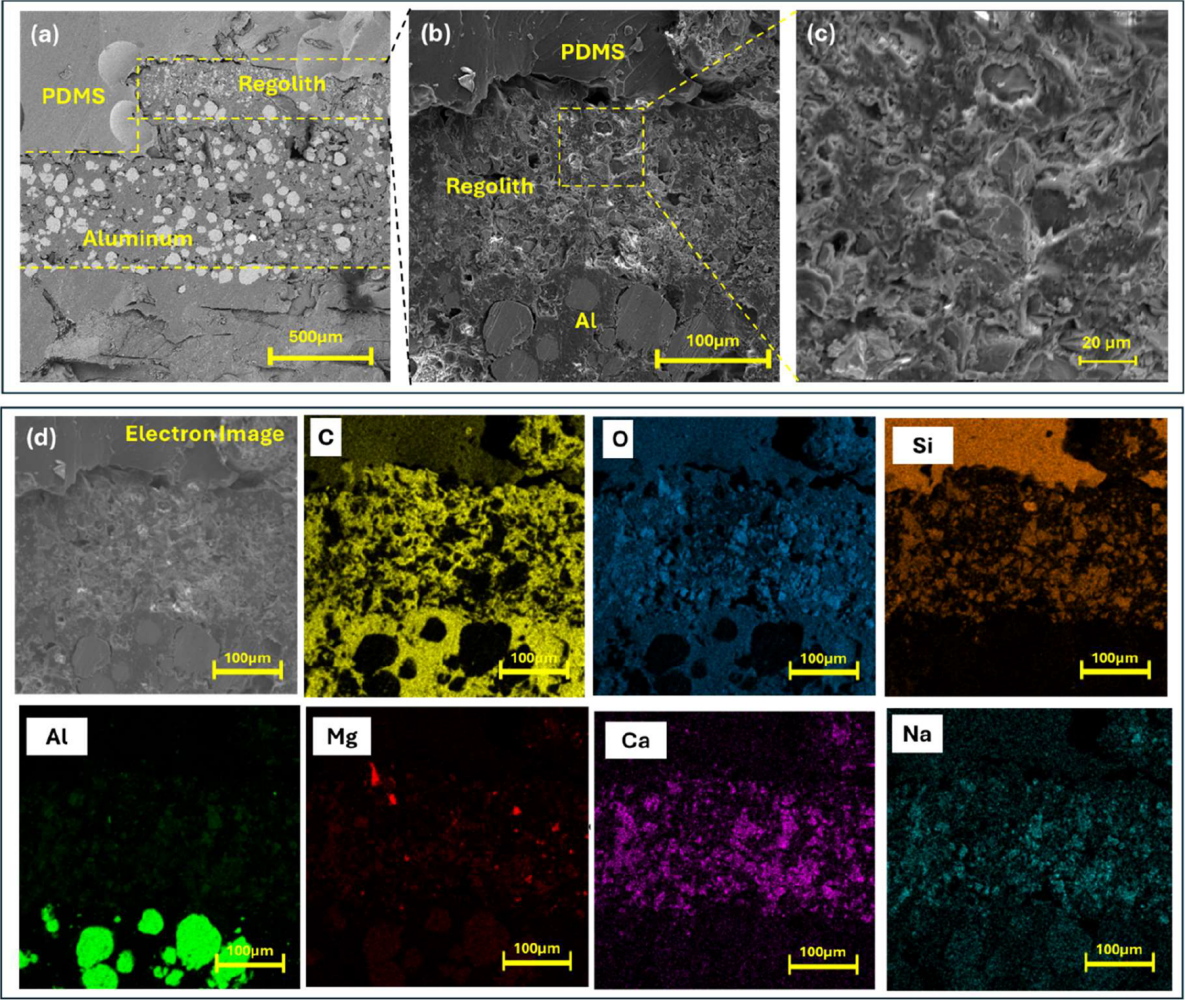
Cross-sectional SEM and EDX characterization of the multimaterial 3D-printed LR/PLA electrode. (a,b) SEM image of the layered device structure consisting of PDMS, LR-containing PLA, and aluminum layer. (c) Higher-magnification view of the selected LR/PLA region. (d) EDX elemental mapping of the cross section across all layers. Scale bars are as indicated.

Furthermore, elemental mapping of the cross section is provided in Figure 3d, which gives more clarity on compositional layers and interfaces. It identified various elements, such as C, O, Si, Al, Fe, Na, Mg, and Ca, in the cross section, in agreement with the oxide-rich mineral composition of lunar regolith simulant. The C and O distribution is observed in all of the layers belonging to PLA, while the contrast between C and O mapping clearly distinguishes the PLA- and regolith-dominant regions. Furthermore, Si mapping dominates in the PDMS layer, and a significant presence of Si in LR/PLA layers also confirms the presence of silicates. The Ca and Na mapping shows a fine presence of LR in the LR/PLA layer. Overall, EDX mapping reveals a clear distribution of elements across the 3D layers with a fused interface, which ensures continuity of charge transfer during TENG operation.

Next, to evaluate the 3D surface morphology of the printed LR/PLA electrode, confocal laser scanning microscopy was performed to obtain quantitative surface roughness data (Supplementary Figure S6). The 3D reconstructed topography reveals a highly uneven and porous structure with multiple micro–meso scale asperities formed by the heterogeneous dispersion of LR particles and local reflow of the PLA matrix during extrusion. The average surface roughness (*R*_a_) was measured to be 8.6 ± 0.5 μm with a root-mean-square roughness (*R*_q_) of 10.3 ± 0.6 μm over a 500 × 500 μm^2^ scan area. Such a rough surface enhances the effective contact area and charge trapping, thereby promoting greater triboelectric charge density and energy output. This quantitative topographic analysis provides direct evidence supporting the role of the LR/PLA porous architecture in improving triboelectric performance.

### 2.2 Triboelectric Performance of LR-Based 3D-TENGs

Figure 4a illustrates the working mechanism of the TENG across its operational stages. The device, constructed to operate via synchronized contact electrification and electrostatic induction, was based on the principles first demonstrated by Wang et al. in their inspiring work on energy harvesting from ambient mechanical sources.^43,44^ According to the mechanism, when materials with higher electron affinity (electronegative) come into contact with those with lower affinity (electropositive), a surface potential difference arises due to a mismatch in their work functions. This interfacial charge transfer, governed by the triboelectric series, aligns with the foundational principles of contact electrification established by Wang et al.^44^ Here, the electronegative triboelectric layer comprises a 3D-printed LR/PLA, while the electropositive layer is an aluminum electrode. A 5-mm-thick polyurethane foam spacer separates the two phases to regulate contact–separation dynamics.^45^

**Figure 4:**
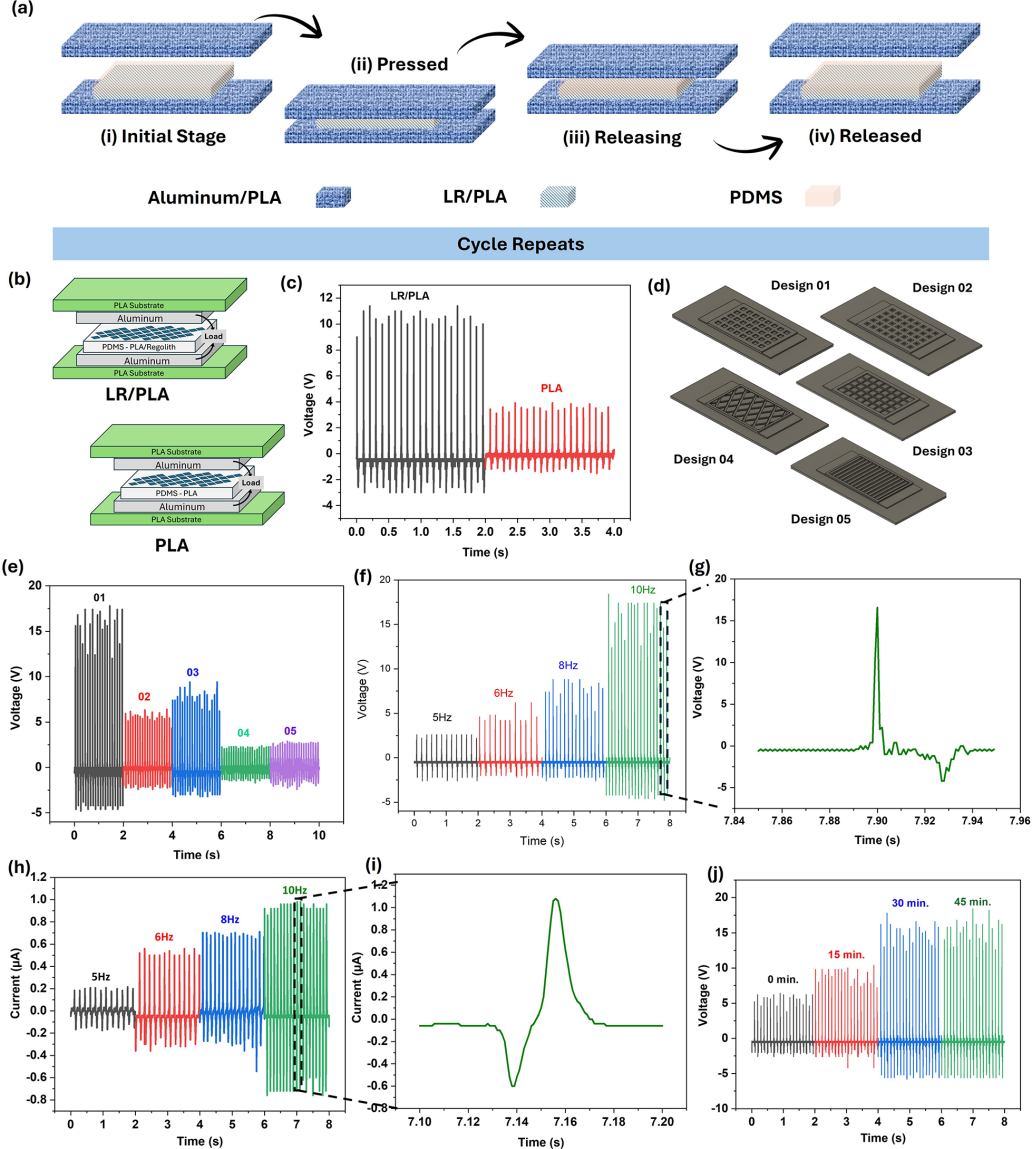
Device operation, structural comparison, design optimization, frequency response, and durability of the 3D-printed TENGs. (a) Schematic illustration of the triboelectric contact–separation cycle, showing the initial state, pressing, releasing, and fully separated stages. (b) Comparison of two TENG architectures: (i) the LR/PLA_PDMS structure and (ii) the PLA_PDMS structure. (c) Open-circuit voltage output for LR/PLA_PDMS compared to PLA_PDMS. (d) Models of five geometric design patterns were evaluated for optimization with an equal mass and a 20 μm depth. (e) Corresponding *V*_oc_ outputs of the designs. (f) Frequency-dependent (*V*_oc_) response measured at 5, 6, 8, and 10 Hz of Design 1. (g) Enlarged representative waveform of the 10 Hz voltage signal. (h) *I*_sc_ performance under the same frequency sweep. (i) Enlarged current signal waveform corresponding to the 10 Hz operation. (j) Durability test over 45 min shows stable output increases at 15 min intervals.

The LR/PLA composite, capped with PDMS, enhances charge density through its porous architecture, which amplifies the interfacial contact area and promotes electron transfer during triboelectrification (Figure 4a, initial stage). This aligns with recent findings by Zhang et al., who established that hierarchical surface structures significantly improve charge accumulation in polymer-based TENGs.^46,47^ Upon mechanical activation via a linear actuator, the aluminum electrode contacts the LR/PLA layer, inducing charge transfer driven by differences in the electron work function (pressing stage). Electrons migrate from aluminum to the composite, generating a triboelectric potential. This phenomenon was quantitatively modeled by Wu et al. using first-principles calculations.^47^ When the surfaces separate (releasing stage), the charge balance is disturbed, causing electrons to flow through the external circuit to neutralize the positive charges on the aluminum layer. This behavior is similar to the charge movement observed in flexible TENGs, as reported by Li et al.^48^ Each time the device is pressed and released, it generates an alternating current. The peak open-circuit voltage (*V*_oc_) and short-circuit current (*I*_sc_) are measured using an oscilloscope (Figure 4b–d). The polyurethane foam acts like a spring, quickly returning the device to its original position after each cycle. This fast recovery reduces energy loss, which is crucial for efficient energy harvesting, as highlighted in recent studies.^49^

To identify the role of LR in TENG performance, two devices are designed as explained in Figure 4b, which are 3D-printed LR/PLA and pure PLA. The peak voltage and current output under different mechanical oscillations are monitored by using a digital oscilloscope. A direct comparison between LR/PLA and PLA TENGs confirms the beneficial effect of LR in PLA, with the composite structure exhibiting nearly 3-fold higher *V*_oc_ (Figure 4c). The LR/PLA device produced a peak voltage of ∼11.2 (±0.2) V, which is much higher than the ∼3.6 (±0.3) V output from the pure PLA particles embedded to the PLA. The roughened and porous structure of the LR/PLA increases the surface area and helps trap more charges, leading to higher voltage generation. In addition, the oxide-rich composition of LR improves the dielectric properties of the composite, enabling more efficient charge storage and reduced recombination. The heterogeneous surface energy of LR also favors a more substantial electron affinity mismatch with the aluminum electrode, which further promotes the interfacial charge transfer. Together, these effects of surface roughness, dielectric enhancement, and favorable material electrode interactions account for the improved performance of LR/PLA compared to pure PLA. The outputs predominantly originate from triboelectric charge transfer between the dielectric electrode and the counter dielectric surface, as evidenced by the contact–separation configuration and the alternating pulse-shaped signals characteristic of TENG operation. These results confirm that the LR/PLA composite is well-suited for energy-harvesting applications.

Further experiments explored how different 3D-printed microstructures of the LR/PLA composite influenced the voltage output. Figure 4d shows various geometric designs tested in this study, each weighing approximately 2.90 g. The study began with square patterns of different sizes (Designs 1–3: small, medium, and large), followed by a diamond-shaped pattern (Design 4) and a grit-shaped pattern (Design 5). All designs featured a porous structure with a depth of 20 μm. Digital images of some printed design electrodes are shown in Figure S7. Corresponding Voc outputs of the five designs are as follows: Design 01 exhibits the highest performance, delivering approximately ∼17.4 (±0.4) V, whereas the remaining designs show ∼5.5 (±0.5), ∼7.8 (±1.0), ∼2.0 (±0.2), and ∼2.7 (±0.2) V, respectively (Figure 4e). Among these, the small square pattern produced the highest voltage output of ∼17.4 (±0.4) V. This improved performance is due to the increased surface area for contact, which leads to better charge distribution during mechanical interactions and collectively boosts mechanical energy harvesting.

To confirm the effective output, testing was carried out using the optimized rectangular patterned design 1 device to evaluate the effect of varying contact frequencies (5, 6, 8, and 10 Hz) on the performance. The results showed that as the contact frequency increased, the voltage output increased simultaneously. We measured a maximum frequency of 10 Hz with the mechanical damping system, yielding a peak output of ∼17.4 (±0.4) V (Figure 4f). This relationship is expected, as a higher contact frequency corresponds to more frequent charge–separation events, thereby increasing energy-harvesting efficiency. To verify the action of a wave during frequency variation, the oscilloscope trace with respect to damping is shown in Figure 4g, which provides a zoomed-in view of the highest wave captured in Figure 4f. Additionally, the current output was analyzed as a function of frequency, with a peak current of ∼0.96 (±0.2) μA recorded at 10 Hz (Figure 4h). This suggests that not only the voltage but also the current output is frequency-dependent, reinforcing the importance of optimizing mechanical excitation in practical applications. This frequency likely corresponds to an optimal balance between contact and separation times, maximizing the charge generation per cycle. An essential aspect of this study was assessing the durability of the TENG device over time. Testing durations ranged from 0 s to 45 min, during which the voltage output increased over time, peaking at ∼17.4 (±0.4) V after approximately 30 min (Figure 4j). This trend suggests that prolonged operation allows for better charge stabilization within the device’s structure. This sustained performance indicates that the TENG device maintains a stable charge transfer rate, likely due to continuous friction between the triboelectric layers, which enhances charge accumulation.

### 2.3 Integration and Applications of LR-Based 3D-TENGs

To study the practical application of the TENG device, a bridge rectifier setup was used to convert the AC output to direct current (DC) (Figure 5a). When tested under varying contact frequencies, the rectified output at 10 Hz produced a peak voltage of ∼17.2 (±0.8) V and a current of ∼1.5 (±0.8) μA (Figure 5b,c). These results demonstrate that the TENG device can deliver stable DC power, which is crucial for charging energy storage devices or powering electronic components in space missions. To simulate real-world conditions, the TENG device was subjected to both regular and vigorous tapping. Regular tapping resulted in a minimum voltage output of ∼9.5 (±0.5) V and a peak of ∼20.8 (±2.0) V, while vigorous tapping produced outputs ranging from ∼17.2 (±3.0) to ∼40 (±2.0) V (Figure 5d). These results demonstrate the device’s ability to harness mechanical energy effectively under varying conditions. This experiment underscores the TENG’s sensitivity to mechanical excitation amplitude, indicating that the device could generate significantly higher outputs under more intense or frequent mechanical stimuli, such as those expected from astronauts’ movements or rover operations on extraterrestrial surfaces. As a final demonstration, the TENG device was used to power the light-emitting diodes (LEDs). Two LEDs were connected to the TENG output during tapping tests (Figure 5e). Both LEDs illuminated brightly, confirming that the generated electricity can be used for practical purposes in space missions, such as powering low-energy devices for sensing and lighting operations (Movie 01).

**Figure 5:**
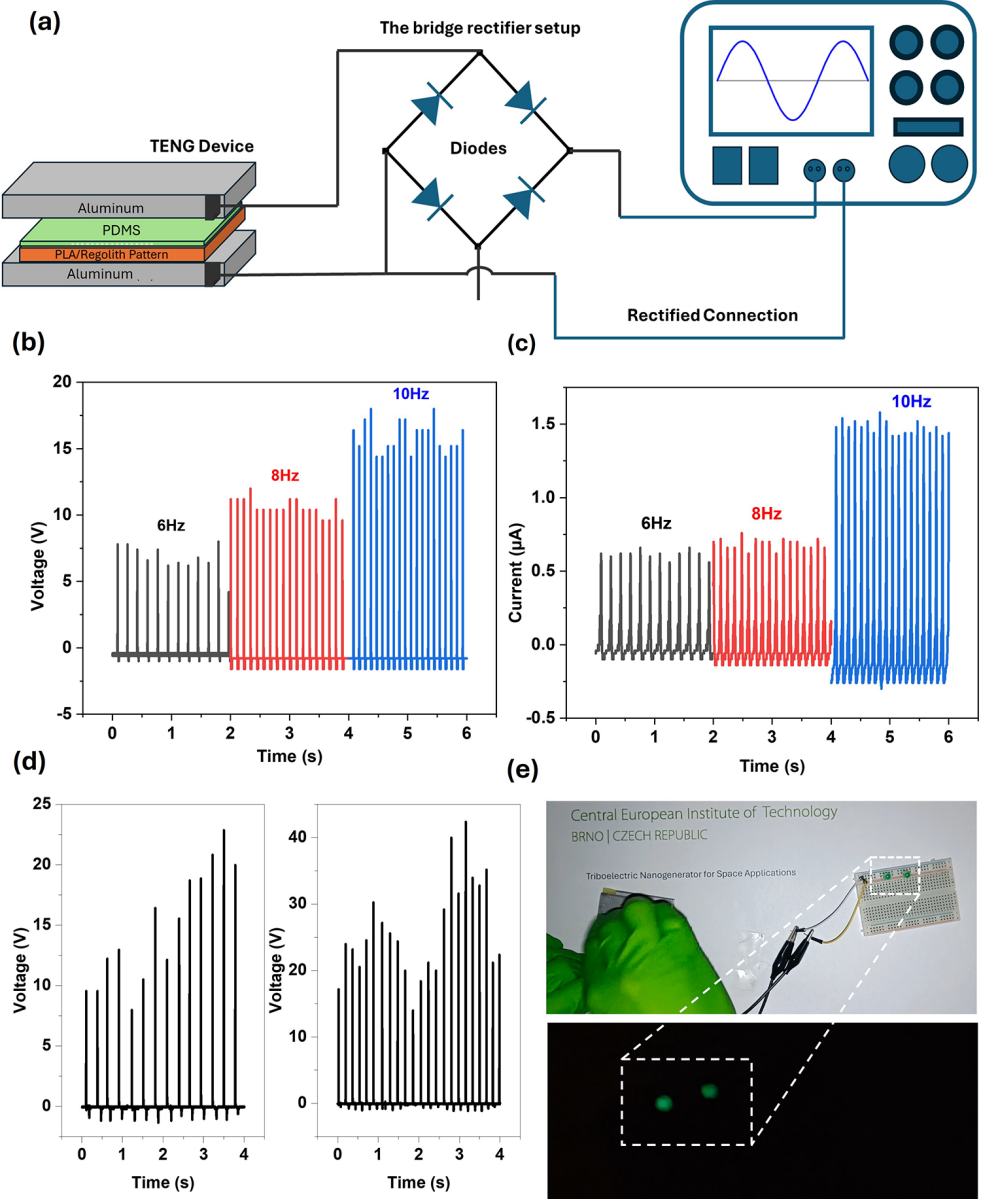
Schematic representation of the experimental setup. (a) Connection of the TENG device with a bridge rectifier and corresponding oscilloscope output. (b,c) Variation of rectified voltage and current at different frequencies while the TENG terminals are connected by a bridge rectifier. (d) Oscilloscope results showing the variation between normal tapping and vigorous tapping on the TENG device. (e) Connection between the TENG device and the LED on the breadboard, with the LED glowing in the dark as shown below.

By attachment of these TENGs to astronaut suits or rover surfaces, the mechanical energy generated by movement can be harvested to power sensors, lights, and other low-power devices. The device’s robust performance under varying mechanical conditions, coupled with its ability to generate both high voltage and current outputs, underscores its viability for sustainable energy harvesting in harsh space environments. However, this TENG design has some limitations due to the weak thermal stability of the polymers. As such, PLA is also biodegradable and susceptible to UV-induced degradation; hence, additional encapsulation or UV-resistant coatings can be implemented in future designs to ensure long-term stability of the device for prolonged space missions, enabling reliable energy harvesting under harsh extraterrestrial conditions. Future devices could be designed with a more robust 3D-printed polymer host to withstand harsh environmental conditions.

## 3 Conclusions

This study demonstrates the potential of 3D-printed LR as the primary material in TENGs devices. Transporting large amounts of materials from Earth to lunar exploration is a major challenge, especially for building a permanent base. By developing TENGs from available LR simulants and combining them with PLA for 3D printing, we pursue a key strategy for future space exploration. We found that the composition of LR with PLA significantly improves the triboelectric performance of bare PLA, due to its favorable negative charge properties. The best performing TENG device with LR/PLA generated a *V*_oc_ of ∼17.4 (±0.4) V and an *I*_sc_ of ∼0.96 (±0.2) μA. By scaling up the device dimensions, the energy output can be significantly enhanced.^50^ In this work, we also confirmed the presence and distribution of regolith within the printed composite through cross-sectional SEM, EDS and XRD analyses, and we used confocal laser scanning to evaluate the surface roughness of the LR/PLA electrode, providing a complete understanding of the material architecture and its role in enhancing triboelectric performance. The experiments were conducted by using mechanically simulated setups, and we also tested the device under real-world conditions by tapping it gently and vigorously. These tests produced voltages of over ∼20.8 (±2.0) V and ∼40 (±2.0) V, respectively, demonstrating the device’s potential for practical applications. This work marks the initial step toward developing TENG devices using LR and 3D-printing integration. This work offers an innovative alternative by converting mechanical energy from LR into electrical power, reducing the reliance on earth-supplied energy sources. This approach not only generates power but also addresses challenges such as static electricity in the Moon’s low-humidity environment. For astronauts, these devices can serve dual purposes: generating energy for powering small devices and acting as environmental sensors and improving safety and operational efficiency.

## 4 Experimental Section

### 4.1 Materials

The Lunar Regolith filament (LR) was procured from Virtual Foundry, USA, under the name *Basalt Moon Dust Filament*. According to the supplier’s technical specifications, the filament consists of a 60–62 wt % lunar regolith simulant blended with a PLA-compliant binder (38–40 wt %). The SYLGARD 184 Silicone Elastomer Kit was obtained from Dow, USA. The PLA filaments for 3D printing were sourced from Filament2Print, Spain.

### 4.2 Preparation of PDMS

The silicone elastomer solution and curing agent were mixed in a 10:1 mass ratio and then stirred for 10 min. The PDMS was kept in a vacuum for 2 h for degassing. Then, the mixture was directly 3D-ink-written with a syringe onto 3D-printed LR/PLA. Finally, the mixture was ready to be placed in an electric oven set at 60 °C for 1.5 h to finish the curing process. The low-temperature curing process of the mixture was carefully chosen to ensure that PLA does not experience distortion. After the preferred period, it was kept at room temperature for 4 h.

### 4.3 Fabrication of the TENG device

The LR/PLA TENG structure was designed using Autodesk Fusion 360 and 3D-printed with a Prusa 3D Printer. The structure was designed and 3D-printed in 3 different steps: a base of 40 mm × 30 mm with Virgin PLA, followed by an aluminum/PLA layer printed at 20 mm × 30 mm, and finally, the LR/PLA rectangular-shaped patterned porous structure was printed onto the top of it with the same dimensions. The prepared PDMS was then printed through direct-ink-writing into the porous structure to complete the bottom electrode. The top electrode was 3D-printed with PLA and coated with aluminum as the counter electrode, featuring a patterned structure. The printing parameters were optimized as follows: a 0.6 mm nozzle, a printing speed of 35 mm/s, and a layer height of 0.2 mm. The bed temperature was maintained at 60 °C, while the nozzle temperature was set to 210 °C for the PLA/LR composite and 230 °C for the aluminum-based filament. Infill density was set to 50% for the triboelectric PLA/LR meshes and 100% for the rest of all layers. Printing was performed in a flat orientation with layer-by-layer deposition to ensure uniform surface morphology. A conductive copper tape with adhesive was attached as a current collector, attaching all the layers and connecting them to the oscilloscope probe terminal. Finally, the top plate was aligned with the bottom plate using polyurethane foam, maintaining a minimum distance of 2 mm between them. After the primary experimental setup, the device was placed under the mechanical damping system and subjected to uniform mechanical actuation of 2.8 N at 10 Hz.

### 4.4 Electrical Measurement

The electrical measurements for this device were recorded by using a digital oscilloscope (GW Instek GDS-1074B). An electric linear motor actuator, referred to as the damping system, with a hub length of 25 mm (24 V DC, max 1000 rpm, DAOE), was used to tap on the TENG. This created a periodic contact release action between the tip of the actuator and the top plate of the TENG device, with frequencies varying from 5 to 10 Hz across all scenarios. The two tips of the oscilloscope probe were connected to the Cu strips of the triboelectric layer and the electrodes on both plates by using a crocodile connector. To achieve the best output results from the oscilloscope, we established an earth connection from the oscilloscope to the damping system. This reduced the noise in the output waveform.

### 4.5 Characterization

The morphology of the 3D-printed electrodes was analyzed using SEM (Mira 3 XMU—Tescan). The elemental mapping of the constituent elements of the samples was carried out with energy-dispersive X-ray spectroscopy (SEM, Mira 3 XMU—Tescan) equipped with an EDS (Oxford Instruments X-MAX) detector. The structural properties of LR/PLA 3D-printed structure were examined via XRD analysis using a Rigaku SmartLab 3 kW X-ray diffractometer running at a voltage of 40 kV and a current of 30 mA. Brag Brentano geometry was employed with Cu Kα radiation (λ = 0.15418 nm). The 3D surface morphology of the 3D-printed LR/PLA electrodes was examined by using a confocal laser scanning microscope.

## Supplementary Material





## Data Availability

The data that support the findings of this study are available at zenodo.org.

## Notes

All-3D-printed LR/PLA-based TENG electrode; XRD spectra of the pure PLA filament; SEM images of the as-received LR/PLA filament before 3D printing; the EDX spectrum and elemental weight percentage of the LR/PLA layer of Figure 3c; EDX elemental mapping of LR/PLA; elemental weight percentage from EDX mapping; CLSM characterization of the 3D-printed LR/PLA electrode; and images of different designs of LR/PLA devices (PDF)
LED powering from continuous tapping on the TENG device (MP4)
