# Supplementary material for: Moon Regolith Simulant-Based All-3D-Printed Triboelectric Nanogenerator for Effective Mechanical Energy Conversion
Source: Energy Fuels. 2026 Feb 4;40(7):3551–61. doi: 10.1021/acs.energyfuels.5c04047 (PMC12926947; doi:10.1021/acs.energyfuels.5c04047)
Supplement: Supplementary file 1 [file ef5c04047_si_001.pdf]

Supporting information

Moon Regolith Simulant-Based All-3D-Printed Triboelectric Nanogenerator for Effective Mechanical Energy Conversion

*Alex Yohannan,[1] Keval K. Sonigara[1], Jayraj V. Vaghasiya,[1] Martin Pumera[1,2,3]**

[1]Future Energy and Innovation Laboratory, Central European Institute of Technology, Brno University of Technology, Purkyňova 123, Brno, 61200 Czech Republic

[2]Faculty of Electrical Engineering and Computer Science, VSB - Technical University of Ostrava, 17. listopadu 2172/15, 70800 Ostrava, Czech Republic

[3]Department of Medical Research, China Medical University Hospital, China Medical University, No. 91 Hsueh-Shih Road, Taichung 40402, Taiwan

*E-mail: pumera.research@gmail.com

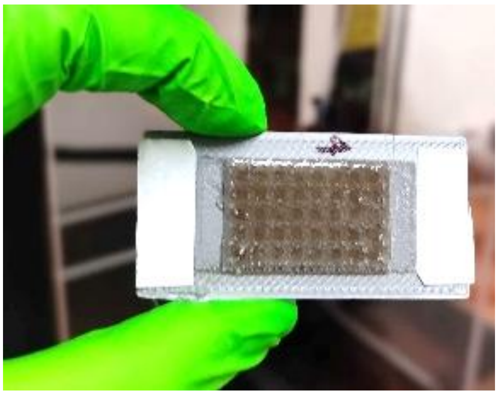

Figure S1. All-3D-printed LR/PLA-based TENG electrode

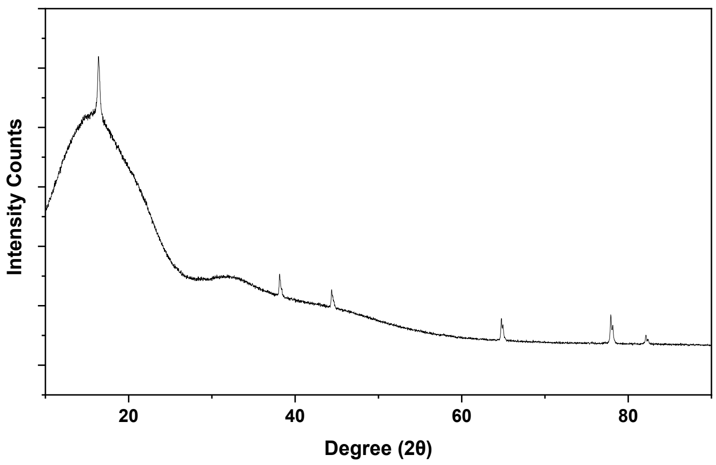

Figure S2. XRD spectra of pure PLA filament

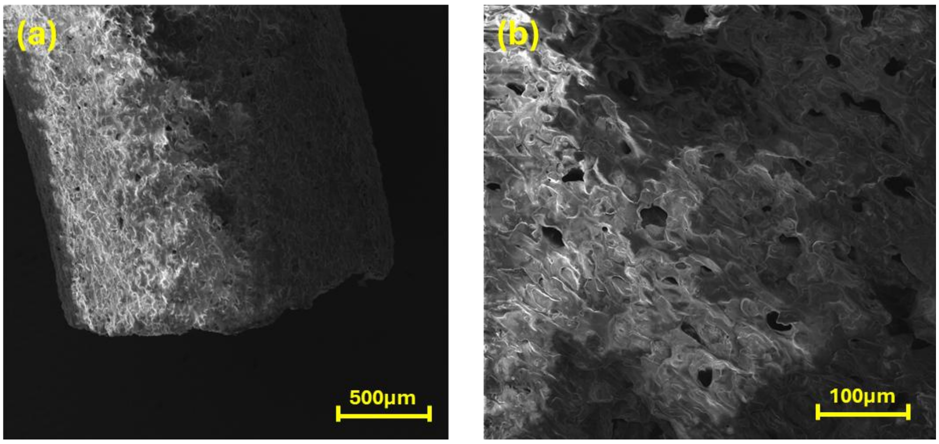

Figure S3. SEM images of the as-received LR/PLA filament before 3D printing. (a) Low-magnification image showing the filament. (b) High-magnification image illustrating surface porosity and evenly distributed LR particles embedded within the PLA matrix.

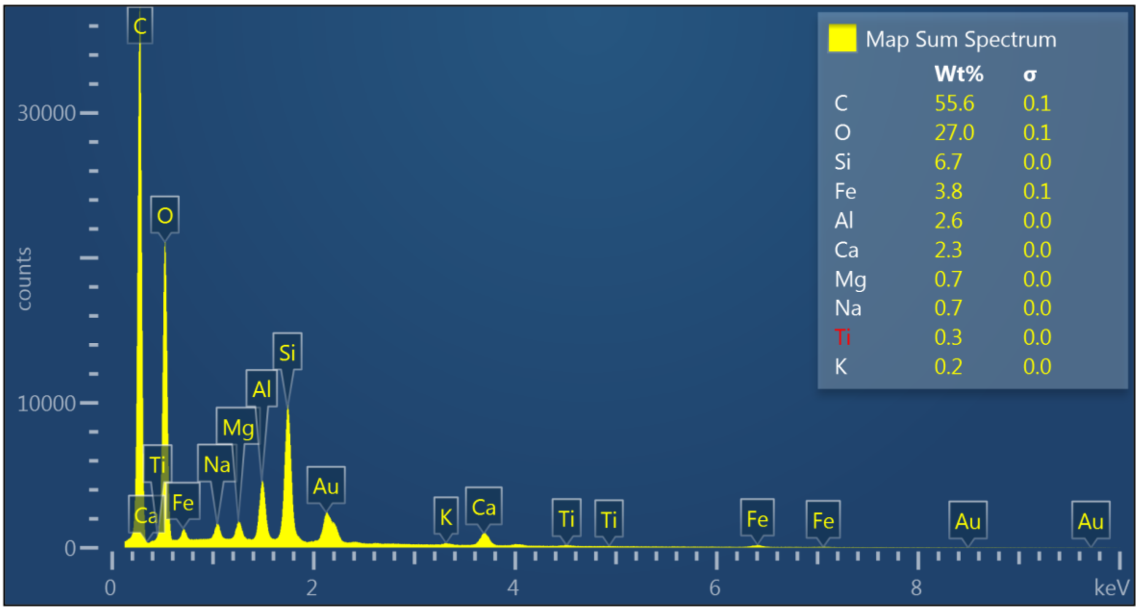

Figure S4. EDX spectrum and elemental weight percentage of LR/PLA layer of Figure 3c.

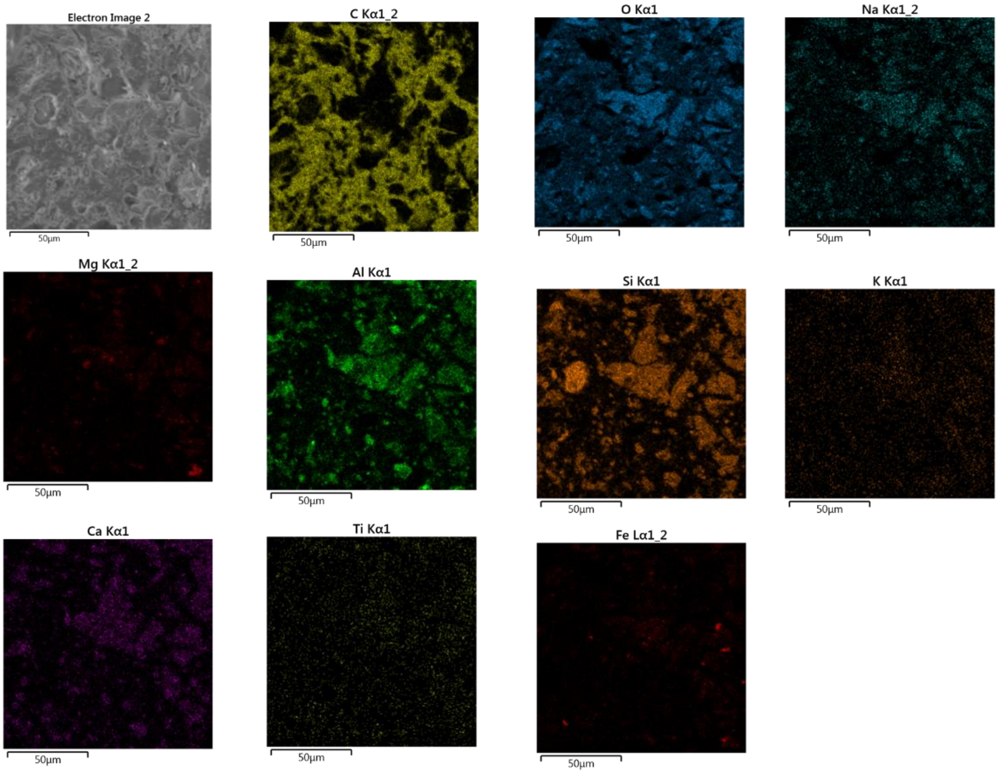

Figure S5. EDX elemental mapping of LR/PLA

Table S1 - Elemental Weight Percentage from EDX Mapping

Element	Wt.%	σ
**C**	55.6	0.1
**O**	27.0	0.1
**Si**	6.7	0.0
**Fe**	3.8	0.1
**Al**	2.6	0.0
**Ca**	2.3	0.0
**Mg**	0.7	0.0
**Na**	0.7	0.0
**Ti**	0.3	0.0
**K**	0.2	0.0

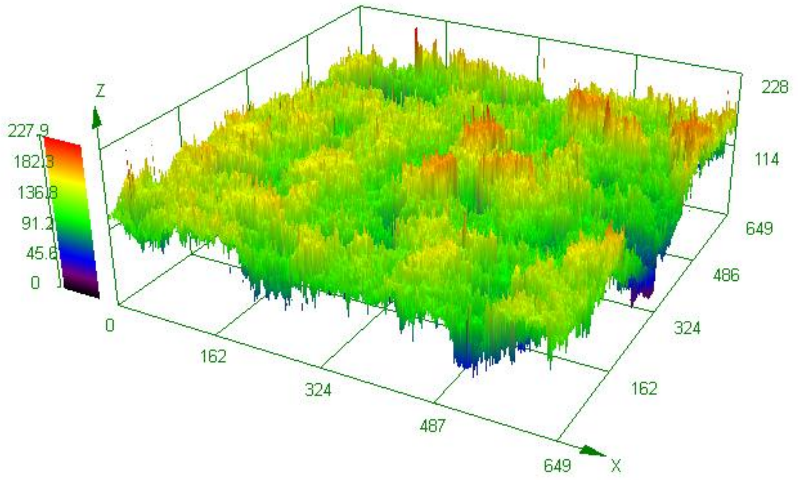

Figure S6. CLSM Characterization of 3D-Printed LR/PLA Electrode

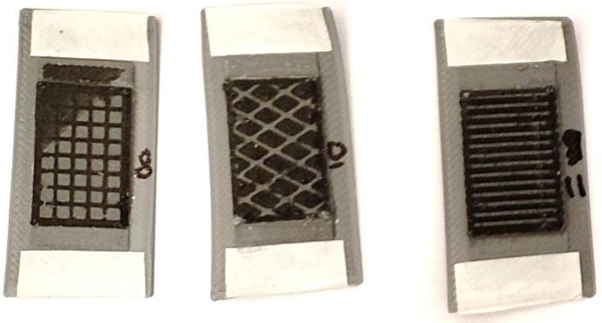

Supplementary Figure S7. Images of different designs of LR/PLA devices.

Supplementary Movie 1. LED powering from continuous tapping on TENG device.
